# Access to Supportive Care Among Australian Cancer Carers: Experiences of Priority Populations

**DOI:** 10.1111/hex.70830

**Published:** 2026-09-09

**Authors:** Stephanie P. Cowdery, Patricia M. Livingston, Thach Tran, Antonina Mikocka‐Walus, Anna Ugalde, Eva Yuen, Hannah Jongebloed, Natalie Winter, Nicole Kiss, Nikki McCaffrey, Norah Refahi, Victoria M. White

**Affiliations:** ^1^ Centre for Quality and Patient Safety Research; Institute for Health Transformation Deakin University Geelong Victoria Australia; ^2^ Office of the Executive Dean, Faculty of Health Deakin University Geelong Victoria Australia; ^3^ School of Psychology, Deakin Lifespan Institute, Faculty of Health Deakin University Burwood Victoria Australia; ^4^ School of Nursing and Midwifery, Faculty of Health Deakin University Geelong Victoria Australia; ^5^ Monash Health Clayton Victoria Australia; ^6^ Institute for Physical Activity and Nutrition Deakin University Geelong Victoria Australia; ^7^ Deakin Health Economics, School of Health and Social Development, Institute for Health Transformation Deakin University

**Keywords:** Australian carers, cancer, caregivers, culturally and linguistically diverse backgrounds CALD, LGBTIQA+, priority populations, rural/remote

## Abstract

**Background:**

Informal carers are essential in supporting people with cancer yet often experience unmet supportive care needs.

**Aims:**

The aim of this study was to examine (a) access to supportive care services (b) perceived barriers to accessing services, and (c) the potential influence of carer health literacy on access and barriers to supportive care among carers across three priority population groups (rural‐remote‐dwelling, LGBTIQA+ and culturally and linguistically diverse (CALD)).

**Methods:**

Cross‐sectional online survey of Australian adult cancer carers. Supportive care use, access barriers, and health literacy (Health Literacy of Caregivers Scale – Cancer (HLSC‐C)) were assessed. Logistic regression examined differences across priority groups, and mediation analyses explored the role of health literacy.

**Results:**

Among 1154 carers, 61.9% accessed some supportive care (46.7% formal services), yet 86.6% reported at least one barrier, with 71.0% reporting two or more. Common barriers were time constraints, service availability, and long wait times. Compared to non‐priority population carers, rural/remote carers had higher odds of reporting limited local availability and services not available when needed (aOR = 5.08 for both). CALD carers had higher odds of reporting a barrier (aOR = 1.81), including long wait times, cost, and lack of culturally appropriate services (aOR = 33.03). Health literacy significantly mediated the association between CALD status and reported barriers.

**Conclusions:**

Cancer carers have limited access to supportive care and face systemic barriers. Inequities remain, particularly for carers from rural/remote areas and those from CALD backgrounds. To improve equity, there is a need for co‐designed, accessible interventions that address health literacy and the diverse geographic, cultural, and linguistic needs of carers.

**Patient or Public Contribution:**

People with lived experience of cancer caregiving were included on this study's advisory committee. In this role, their expertise informed the study design, including the development of the questionnaire, the Participant Information and Consent Form (PICF), and recruitment processes. They were also invited to contribute to data interpretation and the preparation of this manuscript. The lead consumer representative (NR) is listed as a co‐author on this manuscript.

## 1 Background

The global incidence of cancer continues to rise, driven by an aging population, environmental factors, and advances in screening and early detection [1, 2]. Improvements in diagnostic methods and treatment modalities have increased survival rates [1]. In turn, more people receiving treatment for longer periods, including ongoing maintenance therapies and multiple lines of therapy [3]. This has created a growing population living with, and beyond, cancer. At the same time, cancer care has shifted from hospital settings to outpatient and home‐based environments [4] placing significant responsibility on carers, who are often required to manage complex care tasks with limited guidance or support from healthcare professionals [5, 6, 7].

Although many carers report meaningful and rewarding aspects of this role [8], caring is also associated with significant financial, social and emotional costs [9]. High rates of psychological distress, including depression, anxiety and stress, carer burden and social isolation are also documented [10, 11, 12]. These challenges can be further exacerbated for carers from priority populations, including those from rural and remote areas, culturally and linguistically diverse (CALD) backgrounds, and carers from lesbian, gay, bisexual, transsexual, queer, intersex, asexual plus (LGBTIQA + ) communities, who often face additional structural, linguistic, and discriminatory barriers when interacting with healthcare systems [5, 13, 14, 15, 16, 17]. Given the multifaceted and complex demands placed on carers, ensuring equitable access to supportive care which can assist in the prevention and management of the physical, psychological, and social impacts that cancer carers may experience across the disease trajectory, and are critical to improve their quality of life [18, 19].

Carers often find themselves having to drive the process of identifying, making sense of, and navigating relevant information and services intended to support them [20, 21]. Consequently, understanding the types of supportive services accessed by carers, including ad hoc funding for services, as well as barriers impeding access to these services is essential, given that inadequate access to supportive care, likely exacerbates care‑related burden and perpetuates inequities [22]. Health literacy, defined as an individual's capacity to obtain, comprehend, and apply health information in ways that are responsive to both personal needs and broader system contexts [23], is a critical factor in seeking and accessing supportive care. Health literacy is particularly important for carers belonging to priority populations, for whom language barriers, experiences of discrimination, and geographic isolation may further constrain equitable access to information and supportive services [24].

This study aims to examine (a) access to supportive care services (b) perceived barriers to accessing services, and (c) the influence of carer health literacy on access and barriers to supportive care services, among cancer carers, with a focus on carers from priority population groups (rural/remote‐dwelling, LGBTIQA + , and CALD).

## 2 Methods

### 2.1 Study Design

This paper utilises data from a nationwide cross‐sectional survey of Australian carers of people with cancer. The survey, designed to explore the experiences and unmet needs of carers, was conducted online via the secure online platform Qualtrics©, between 24 June 2024, and 31 January 2025. To enhance data integrity, the online survey included a CAPTCHA verification mechanism and survey completion times were assessed as part of the data screening process to identify potentially invalid or low‐quality responses. Full details on the study design and methodology have been published elsewhere [25].

### 2.2 Eligibility

Eligible participants were Australian residents, aged 18+ years, who had, or were, providing informal care for someone with cancer (aged ≥ 15 years at time of diagnosis) within the past 5 years, and had access to an internet connected device.

### 2.3 Recruitment

Participants were recruited via two approaches. First, through paid and unpaid social media advertisements (e.g. Facebook, Instagram), alongside promotion via cancer and carer organisations, hospitals, LGBTIQA+ healthcare clinics, and consumer groups. Second, the LOTE Agency^1^ used its database and community connections to recruit carers from CALD communities who spoke Mandarin, Cantonese, Vietnamese, Arabic, and/or Punjabi at home. All materials, including advertisements, screening, and surveys, were available in these five languages, with professional translation by LOTE.

Interested participants completed online screening questions to determine eligibility before being automatically directed to the full survey.

### 2.4 Outcome Measures

#### 2.4.1 Access to Supportive Care Services

Data on uptake of supportive care were obtained via the open‐ended question ‘*What organisations, websites, or other services have you primarily accessed to seek support for yourself in your caring role?*’.

Verbatim responses were systematically coded independently by two researchers [SC and ST] into distinct categories. Discrepancies were resolved through consultation with a third, independent researcher. Carers were able to be assigned to multiple categories. Formal service categories included: (i) Cancer organisations (e.g. cancer council(s), Breast Cancer Network Australia, Leukaemia Foundation, etc.), (ii) health and medical services (e.g. hospital/health service, medical professionals, allied health, palliative care, etc), (iii) mental health and counselling (e.g. psychology or counselling services, support and social workers, yoga/meditation/mindfulness), (iv) carer services (e.g., Carers Gateway, Carers Australia), and v) government and community services (e.g., Centrelink, community transport services, aged care). Informal service categories included: (i) information and digital services (e.g., internet/Google searches and social media platforms), and (ii) informal social networks (e.g., family and friends, church or community group).

#### 2.4.2 Perceived Barriers to Accessing Supportive Care Services

To explore potential barriers in accessing supportive care services, participants were asked: ‘*Thinking about your experiences supporting someone with cancer, have you experienced any of the following challenges with accessing services to support you? (tick all that apply)*’. The development of these items was based on the National Carer survey [8]. Ten predefined barriers spanning both system‑level and individual‑level domains were presented, along with an open‑ended ‘Other (please specify)’ response option (see Table 2).

#### 2.4.3 Health Literacy

The Health Literacy of Caregivers Scale–Cancer (HLSC‐C) is a 46‐item scale used to assess health literacy in the context of supporting someone with cancer [26]. The scale evaluates carers' ability to access, understand, appraise, and apply health information relevant to their caregiving role. The scale includes ten domains that comprise carer health literacy, identified through nominal group techniques (see Table 2 for domain descriptions). For eight domains, participants indicated strength of agreement or disagreement (4 = point scale) with responses then recoded as strongly agree/agree = 1; and disagree/strongly disagree = 0. For two domains, respondents were asked to ‘Please indicate how easy or difficult the following tasks are for you to do now’, using a 5‐point Likert scale (cannot do; very difficult; quite difficult; quite easy; or very easy). Response options were recoded: ‘cannot do'/‘very difficult'/‘quite difficult' = 0; and ‘quite easy'/‘very easy' = 1. Mean scores for each domain are reported, with higher scores suggesting greater health literacy capacity in that domain. HLCS‐C domains have shown adequate internal consistency (0.78‐0.92) [26], and for the current study, ranged from 0.63 to 0.85.

### 2.5 Carer Priority Group Membership

The following priority population groups were included in analyses. Participants could belong to more than one priority group.

### 2.6 Carers from Rural/Remote Areas

Carers residential postcode was used to define regionality using the Modified Monash (MM) Model 2023 edition [27]. This model categorises postcodes into seven categories reflecting increasing rurality and remoteness. For the present study, categories were dichotomised as metropolitan areas/large regional centres (MM1‐MM2) and rural/remote areas (MM3‐MM7) [28], with the second group forming this priority group.

### 2.7 Carers Identifying As LGBTIQA+ 

Carers were identified as LGBTIQA+ through the question ‘Do you consider yourself to be: ‘Heterosexual or straight’, ‘Lesbian, Gay or Homosexual’, ‘Bisexual’, ‘Transexual’, ‘Queer’, ‘Intersex’, Asexual’, ‘prefer not to answer’ with a free‐text response option provided to allow self‐description.

### 2.8 Carers from Culturally and Linguistically Diverse Backgrounds (CALD)

CALD carers were identified through the question ‘Do you speak or use a language other than English at home?’, with those indicating ‘Yes’ asked to nominate their primary spoken language(s).

### 2.9 Demographic and Care Characteristics

The questionnaire gathered information on both the carer's and care recipient's demographic details, including clinical characteristics. All items were self‐reported and are described in full elsewhere [25].

### 2.10 Analyses

Data were managed and analysed using Stata version 19 (StataCorp, College Station, TX, USA).

Participant characteristics and study outcomes were summarised using means and standard deviations for scores and frequency and percentages for categories. Differences in study outcomes for each priority group (rural/remote vs non‐rural/remote, CALD vs non‐CALD, and LGBTIQA+ vs non‐LGBTIQA + ), were explored with chi‐square for categorical variables and tests for continuous data. The mechanism of missing data was examined by comparing the main characteristics of participants included in the analyses with those of participants excluded from the analyses and Little's chi‐squared test for Missing Completely At Random (MCAR) [29].

Logistic regression models were fitted to examine relative associations between membership in each priority group (rural/remote, CALD, and LGBTIQA + ) and each outcome. Specifically, access to supportive care services (any supportive care accessed, formal supports accessed, and informal supports accessed (all yes vs. no)) and perceived barriers to accessing supportive care services (any barriers, and for each of the individual 10 prespecified barriers (yes vs. no)). Models tested the impact of belonging to each priority group compared to not belonging to any priority group after adjusting for potential confounders of age (18–49 years [referent], 50–59 years, 60+ years), gender (female [referent], male) employment status (not in paid employment [referent], in paid employment), education (year 12 certificate or less [referent], certificate/diploma, bachelor's degree or higher), presence of a long‐term health condition (no [referent], yes), duration of care ( = < 1 year [referent], ≥ 1 year <–to 3 years, ≥ 3 years< to 5 years, ≥ 5 years), and support of care recipient with palliative care (no [referent], yes), selected a priori. Results were presented as adjusted odds ratios (aOR), 95% confidence intervals (95% CI) and *p*‐value for significance.

Missing data were handled using a pairwise deletion approach. Sensitivity analyses were conducted to examine the impact of missing data using multiple imputation. The imputation model included all study variables used in the main analysis. A total of 30 imputed datasets were created using the mi impute chained procedure in Stata [30], and parameter estimates were combined using Rubin's rules [31]. Interaction effects between priority population characteristics were not examined, as the sample sizes within several priority population groups were insufficient to support robust intersectional analyses.

Model calibration was assessed using the Hosmer–Lemeshow goodness‐of‐fit test, with *p* > 0.05 indicating no evidence of poor fit and therefore adequate calibration. Multicollinearity was assessed using the Variance Inflation Factor (VIF). Variables with VIF > 5 were considered to exhibit problematic multicollinearity and were excluded from the final models. Potentially influential observations were identified by examining standardised residuals and Pregibon's Dbeta statistic following logistic regression. These observations were first assessed to determine whether they resulted from data errors. The models were then refitted after excluding influential observations to evaluate the robustness of the findings. Any substantial change (> 20%) in the coefficient of the primary exposure variable (priority group) was noted and discussed.

Causal mediation analysis was conducted to examine the extent to which health literacy mediates the associations between membership in each priority group (rural/remote, culturally and linguistically diverse [CALD], and LGBTIQA + ) and each outcome. Analyses were performed using the mediate command in Stata. For these analyses, we specified a mediation model in which priority group membership was treated as the exposure, health literacy the mediator, and supportive care use/reported access barriers the outcomes. Natural indirect effects (mediation effects), natural direct effects, and total effects were estimated within a counterfactual (potential outcomes) framework. All models were adjusted for potential confounders of the exposure–outcome, exposure–mediator, and mediator–outcome relationships, as in the main models. Standard errors and 95% confidence intervals were estimated using the delta method. Results were presented as adjusted odds ratios (aOR), 95% confidence intervals (95% CI) and p‐value for significance. A two‐sided alpha level of 0.05 was used to determine statistical significance for all tests.

### 2.11 Ethical Approval

Ethics approval was obtained from the Human Research Ethics Committee at Deakin University (HEAG‐H 34‐2024).

## 3 Results

### 3.1 Participant Characteristics

Of the 1,536 participants who took part in the survey, 382 were excluded from this analysis as they did not complete the relevant survey questions and had missing data for the primary outcomes.

In total, 1,154 participants (75.1%), completed all study questions and were included in this analysis. Most carers were female (82.1%, *n* = 947), aged 50 years or over (62.8%, *n* = 725), and more than half held a bachelor's degree or higher (52.3%, n = 604), and were in paid employment (57.4%, *n* = 662). Around one‐third resided in a rural/remote area (28.8%, *n* = 332) or were from a CALD background (28.1%, *n* = 324), with 9.5% (*n* = 110) identifying as LGBTIQA + ; approximately 40.5% (n = 467), did not describe being part of a priority population. Demographic and care characteristics between participants included versus excluded from analyses are described in Supporting Information: Table S1. Several characteristics of participants excluded from the analyses differed significantly from those of participants included in the analyses (Supporting Information: Table S1). The proportions of missing data for the major variables among included participants were all < 3% and the p‐value of the Little's chi‐squared test for MCAR < 0.001 (Supporting Information: Table S2). Overall, missingness was likely related to observed variables (MAR).

### 3.2 Access to Supportive Care Services and Reported Barriers to Accessing Supportive Care Services

#### 3.2.1 All Carers

Access to supportive care services and reported barriers to using services are described in Table 1. Overall, 61.9% (*n* = 714) of all carers reported accessing some form of support for themselves in their care role. Around half (46.7%, *n* = 539) of all carers, reported accessing formal support, most commonly cancer organisations (24.4%, *n* = 281), and health and medical services (19.5%, *n* = 225). Informal support included information and digital services (10.9%, *n* = 126), and participants social networks (5.4%, *n* = 62).

**Table 1:** Access to supportive care and reported barriers to accessing supportive care services among carers overall and by priority population group membership status (*n*%).

	Total sample (*n* = 1154)		Priority population group membership							
			Non‐member		Rural/remote		CALD		LGBTIQA+	
			(*n* = 467)		(*n* = 332)		(*n* = 324)		(*n* = 110)	
	*n*	%	*n*	%	*n*	%	*n*	%	*n*	%
*Access to supportive care services*										
Supportive care [any] accessed (yes)	714	61.9	288	61.7	212	63.9	200	61.7	60	54.5
Formal supportive care (yes)^b^	539	46.7	218	46.7	153	46.1	158	48.8	48	43.6
Cancer specific organisations	281	24.4	115	24.6	89	26.8	68	21.0	25	22.7
Health and medical services	225	19.5	99	21.2	66	19.9	61	18.8	14	12.7
Mental health and counselling^b^	112	9.7	52	11.1	38	11.4	19	5.9	12	10.9
Carer specific organisations	92	8.0	42	9.0	28	8.4	18	5.6	8	7.3
Government and community services	62	5.4	25	5.4	19	5.7	16	4.9	3	2.7
Informal Supportive care (yes)	175	15.2	70	15.0	37	11.1	42	13.0	12	10.9
Information and digital services	126	10.9	49	10.5	59	17.8	37	11.4	10	9.1
Informal support networks^a,b^	62	5.4	24	5.1	28	8.4	8	2.5	4	3.6
*Barriers to accessing supportive care services*										
Nil Barriers	151	13.1	75	16.1	40	12.0	30	9.3	12	10.9
Barriers (yes)^b^	999	86.6	390	83.5	292	88.0	292	90.1	98	89.1
1 Barrier	180	15.6	94	20.1	41	12.3	43	13.3	14	12.7
2–3 Barriers	415	36.0	163	34.9	116	34.9	129	39.8	39	35.5
4‐5 Barriers	277	24.0	103	22.1	74	22.3	86	26.5	34	30.9
6+ Barriers^a^	127	11.0	30	6.4	61	18.4	34	10.5	11	10.0
Barriers Specified										
Time and energy needed to organise services^a,b^	536	46.4	220	47.1	172	51.8	119	36.7	59	53.6
Lack of time to seek out support or services for myself^a,b^	492	42.6	203	43.5	160	48.2	105	32.4	57	51.8
Long waiting periods before you can access services	456	39.5	150	32.1	137	41.3	157	48.5	48	43.6
Services not being available locally^a^	427	37.0	116	24.8	204	61.4	108	33.3	35	31.8
Services not available when I needed them^a^	422	36.6	150	32.1	140	42.2	126	38.9	38	34.5
Finding information about services available to me is difficult^b^	361	31.3	156	33.4	106	31.9	83	25.6	33	30.0
High cost of services mean they are unaffordable^b^	264	22.9	82	17.6	66	19.9	107	33.0	30	27.3
No services appropriate for cultural background^a,b^	93	8.1	4	0.9	7	2.1	88	27.2	9	8.2
No services appropriate for my gender identity^a,b^	48	4.2	2	0.4	5	1.5	41	12.7	5	4.5
No services appropriate for my sexual identity^b,c^	43	3.7	0	0.0	3	0.9	36	11.1	10	9.1
Other	153	13.3	64	13.7	63	19.0	17	5.2	15	13.6

*Note:* Missing data: Barriers *n* = 4. Significant difference on chi‐square (*p* < 0.05) for ^a^rural/remote vs. metro, ^b^CALD vs. non‐CALD, and ^c^LGBTIQA+ vs. non‐LGBTIQA+.

Almost all carers (86.6%, *n* = 999) reported experiencing barriers to accessing supportive care services. The most common barriers included the time and energy needed to organise services (46.6%, *n* = 536), lack of time to seek out support or services for [themselves](42.6%, *n* = 492), long wait periods before [they] can access services (39.5%, *n* = 456), and services not being available locally (37.0%, *n* = 427), or when needed (36.6%, *n* = 422). Around one third reported that finding information about services was difficult (31.3%), and almost one‐quarter (22.9%) reported the cost of services made them unaffordable. Experiencing multiple barriers was common, with 36.0% reporting two or three barriers, 24.0% reporting four or five barriers, and 11.0% reporting six or more barriers to accessing supportive care for themselves.

#### 3.2.2 Priority Population Group Carers

For rural/remote carers, a higher percentage reported using informal support networks compared to metropolitan carers (8.4% vs. 4.2%). For barriers to accessing supportive care, a higher percentage of rural/remote carers reported facing multiple barriers (6+ barriers 18.4% vs. 7.8%), plus the time and energy needed to organise services (51.8 vs. 44.8), lack of time to seek out support or services (48.2% vs. 40.1%), and services not being available locally (61.5% vs. 26.8%), or when needed (42.2 vs. 34.4%,) compared to metropolitan carers (all *p* < 0.05).

Among CALD carers, while a higher percentage reported accessing formal support services compared to non‐CALD (63.5% vs. 52.6%), a lower percentage accessed mental health and counselling services (5.9% vs. 11.2%) or informal support networks (2.5% vs. 6.5%). A higher percentage of CALD carers reported facing barriers [any] to supportive care (90.7% vs. 85.4%), as well as long waiting periods (48.5% vs. 36.0%), high cost (33.0% vs. 18.8%), difficulty finding information (25.6% vs. 33.4%), and reporting services weren't appropriate for [their] cultural background (27.2% vs. 1.0%), or gender (12.7% vs. 1.0%) or sexual identity (11.1% vs. 1.0%). A lower percentage reported the time and energy needed to organise services (36.7% vs. 50.2%), and lack of time to seek out support (32.4% vs. 46.6%), compared to non‐CALD carers (all *p* < 0.05).

For LGBTIQA+ carers, a higher percentage reported barriers to accessing support including lack of time to seek out support (51.8% vs. 41.8%), and that services were not appropriate for [their] sexual identity (9.1% vs. 3.2%), compared to non‐LGBTIQA+ carers (both *p* < 0.05).

### 3.3 Health Literacy

#### 3.3.1 All Carers

The distribution of health literacy scores is described in Table 2. The mean health literacy total score for carers in this sample was 25.61 (SD = 9.07). The domains with the highest scores (which represents higher understanding or greater ease), were: Understanding care recipient needs and preferences (*M* = 4.36 [SD 1.72]), Understanding the healthcare system (*M* = 3.23 [1.62]), and Cancer‐related communication with the care recipient (*M* = 2.96 [1.29]). The lowest scores were observed for Social Support (*M* = 1.45 [0.76]), Self Care (*M* = 1.89 [1.69]), and Proactivity and determination to seek information (*M* = 2.08 [1.07]).

**Table 2:** Distribution of health literacy scores among carers overall and by priority population group membership status presented as mean and standard deviation (M [SD]).

	Total sample (*n* = 1154)		Priority population group membership							
			Non‐member		Rural/remote		CALD		LGBTIQA+	
			(*n* = 467)		(*n* = 332)		(*n* = 324)		(*n* = 110)	
	M	SD	M	SD	M	SD	M	SD	M	SD
Health literacy										
Total Score^a,b^	25.61	9.07	26.38	8.66	**26.94**	**8.73**	**23.00**	**9.55**	24.89	9.63
Domains										
Domain 1. Proactivity and determination to seek information (3 items)^a,b^	2.08	1.07	2.07	1.08	**2.24**	**1.00**	**1.89**	**1.10**	2.04	1.06
Domain 2. Adequate information about cancer and cancer management (4 items)^a,b^	2.29	1.45	2.35	1.47	**2.45**	**1.45**	**1.98**	**1.36**	2.14	1.41
Domain 3. Supported by healthcare providers to understand information (4 items)^a,b^	2.37	1.48	2.42	1.52	**2.56**	**1.49**	**2.18**	**1.37**	2.22	1.47
Domain 4. Social support (2 items)^b,c^	1.45	0.76	1.57	0.71	1.48	0.73	**1.29**	**0.79**	**1.28**	**0.83**
Domain 5. Cancer‐related communication with the care recipient (4 items)^a,b^	2.96	1.29	3.10	1.23	**3.25**	**1.13**	**2.44**	**1.37**	2.91	1.28
Domain 6. Understanding care recipient needs and preferences (6 items)^a,b^	4.36	1.72	4.52	1.60	**4.74**	**1.58**	**3.68**	**1.89**	4.23	1.81
Domain 7. Self‐care (5 items)^a,b^	1.89	1.69	1.81	1.72	**1.60**	**1.56**	**2.35**	**1.63**	1.92	1.70
Domain 8. Understanding the healthcare system (5 items)^a,b^	3.23	1.62	3.33	1.62	**3.40**	**1.60**	**2.83**	**1.63**	3.06	1.63
Domain 9. Processing health information (4 items)^b^	2.48	1.58	2.56	1.63	2.57	1.63	**2.18**	**1.44**	2.59	1.50
Domain 10. Active engagement with healthcare providers (4 items)^b^	2.51	1.60	2.56	1.65	2.65	1.63	**2.31**	**1.46**	2.47	1.59

*Note:* Missing data: Health literacy total *n* = 25, domain 1 *n* = 5, domain 2 *n* = 5, domain 3 *n* = 6, domain 4 *n* = 4, domain 5 *n* = 6, domain 6 *n* = 9, domain 7 *n* = 7, domain 8 *n* = 9, domain 9 *n* = 3, domain 10 *n* = 5. The Cronbach's alpha for each domain were: domain 1 α = 0.66, domain 2 α = 0.72, domain 3 α = 0.76, domain 4 α = 0.63, domain 5 α = 0.72, domain 6 α = 0.74, domain 7 α = 0.74, domain 8 α = 0.73, domain 9 α = 0.83, and domain 10 α = 0.85. Significant difference on *t*‐test (*p* < 0.05) for ^a^rural/remote vs. metro, ^b^CALD vs. non‐CALD, and ^c^LGBTIQA+ vs. non‐LGBTIQA+. Bold values are significant difference on *t*‐test/logistic regression/mediation analysis *p* < 0.05.

#### 3.3.2 Priority Population Group Carers

Rural‐remote carers had a higher mean total health literacy score than metropolitan carers (26.91 [8.78], vs. 25.21 [9.09], *p* < 0.004). A higher mean score was observed for proactivity and determination to seek information (2.24 [0.99], vs. 2.02 [1.08]), adequate information about cancer (2.44 [1.46], vs. 2.23 [1.43]), supported by healthcare providers to understand information (2.53 [1.51], vs. 2.32 [1.47]), cancer‐related communication with the care recipient (3.25 [1.13], vs. 2.87 [1.31]), understanding care recipient needs (4.72 [1.59], vs. 4.24 [1.75]), and understanding the healthcare system (3.40 [1.58], vs. 3.18 [1.62]), (all *p* < 0.05). With a lower mean score for self‐care (1.59 [1.56], vs. 2.00 [1.72], *p* < 0.05).

Carers from CALD background had a significantly lower mean health literacy total score (22.86 [9.58]), compared to non‐CALD carers (26.61 [8.67], *p* < 0.001), and scored lower across almost all domains (all *p* < 0.05). Specifically, proactivity and determination to seek information (1.89 [1.11], vs. 2.15 [1.04]), adequate information about cancer (1.97 [1.36], vs. 2.41 [1.46]), supported by healthcare providers to understand information (2.17 [1.38], vs. 2.46 [1.51]), social support (1.27 [0.79], vs. 1.52 [0.73]), cancer‐related communication with the care recipient (2.44 [1.37], vs. 3.16 [1.19]), understanding care recipient needs (3.66 [1.89], vs. 4.63 [1.58]), understanding the healthcare system (2.82 [1.63], vs. 3.38 [1.59]), processing health information (2.16 [1.44], vs. 2.60 [1.62]), and active engagement with healthcare providers (2.29 [1.47], vs. 2.58 [1.65]). Though, scored higher on self‐care (2.35 [1.64], vs. 1.72 [1.67]).

LGBTIQA+ cares had a lower mean score for the domain of social support (1.29 [0.83]), compared to non‐LGBTIQA+ carers (1.47 [0.75], *p* < 0.05).

### 3.4 Likelihood of Accessing Support Services (Any, Formal and Informal), and Barriers to Across Carer Priority Populations

Table 3 presents adjusted odds ratios (aOR) for the associations between carer priority population group (rural/remote, CALD, or LGBTIQA+ vs non‐priority group) and i) access to supportive care services (any, formal and informal), and ii) barriers to accessing supportive care services. Carers from rural/remote areas had a higher odds of reporting select barriers to accessing supportive care for themselves compared to carers who were not part of any priority group. Specifically, there were higher odds for both reporting services were not locally available (aOR = 5.08 95% CI 3.75; 6.87] *p* < 0.001), or available when needed (aOR = 1.52 95% CI 1.13; 2.03 *p* = 0.006).

**Table 3:** Logistic regression models predicting odds of (i) accessing supportive care services (any, formal and informal), and (ii) reporting barriers to accessing supportive care for each carer priority population group of interest compared to carers not from any priority population group.

	*N*	Rural/remote (ref: non‐priority group carers)			CALD (ref: non‐priority group carers)			LGBTIQA+ (ref: non‐priority group carers)		
		aOR	[95% CI]	*p* value	aOR	[95% CI]	*p* value	aOR	[95% CI]	*p* value
*Access to supportive care services*										
Supportive care [any] accessed (yes)	1060	1.08	[0.81; 1.44]	0.621	0.94	[0.66; 1.34]	0.731	0.67	[0.423; 1.03]	0.070
Formal supportive care (yes)	1060	0.90	[0.67; 1.22]	0.508	1.41	[0.96; 2.07]	0.080	0.88	[0.54; 1.43]	0.601
Informal supportive care (yes)	1060	1.21	[0.82; 1.78]	0.331	0.75	[0.45; 1.25]	0.273	0.69	[0.34; 1.40]	0.301
*Barriers to accessing supportive care services*										
Barriers [any] vs none	1056	1.40	[0.92; 2.13]	0.122	**1.81**	**[1.03; 3.16]**	**0.038**	0.83	[0.42; 1.63]	0.589
Long waiting periods before you can access services (yes vs no)	1056	1.33	[0.99; 1.78]	0.058	**1.77**	**[1.25; 2.52]**	**0.001**	0.98	[0.63; 1.54]	0.941
Services not being available locally (yes vs no)	1060	**5.08**	**[3.75; 6.87]**	**< 0.001**	1.12	[0.77; 1.63]	0.561	0.65	[0.39; 1.08]	0.099
Services not available when I needed them (yes vs no)	1060	**1.52**	**[1.13; 2.03]**	**0.006**	**1.58**	**[1.10; 2.26]**	**0.013**	0.75	[0.47; 1.21]	0.244
No services appropriate for cultural background (yes vs no)	1060	0.66	[0.25; 1.74]	0.405	**33.03**	**[12.18; 89.57]**	**< 0.001**	0.70	[0.27; 1.83]	0.466
No services appropriate for my gender identity (yes vs no)	1060	1.14	[0.39; 3.37]	0.814	**11.14**	**[3.66; 33.93]**	**< 0.001**	0.88	[0.25; 3.15]	0.848
No services appropriate for my sexual identity (yes vs no)	1060	1.05	[0.6; 4.30]	0.944	**9.22**	**[2.64; 32.25]**	**0.001**	2.75	[0.90; 8.38]	0.075
High cost of services mean they are unaffordable (yes vs no)	1060	1.08	[0.76; 1.54]	0.682	**1.80**	**[1.21; 2.69]**	**0.004**	0.89	[0.53; 1.50]	0.651
Finding information about services available to me is difficult (yes vs no)	1060	0.91	[0.67; 1.22]	0.510	0.74	[0.51; 1.08]	0.120	1.04	[0.65; 1.66]	0.887
Time and energy needed to organise services (yes vs no)	1060	1.30	[0.97; 1.73]	0.078	0.78	[0.55; 1.11]	0.163	1.05	[0.67; 1.66]	0.820
Lack of time to seek out support or services for myself (yes vs no)	1060	1.32	[0.99; 1.75]	0.058	0.75	[0.53; 1.07]	0.117	1.28	[0.81; 2.00]	0.287

*Note:* Models controling for: Carer characteristics: age (18–49 (ref); 50–60 + ), gender (female (ref) male), employment (not in paid employment (ref), in paid employment), education (<=Year 12 (ref); certificate/diploma; Bachelor + ), presence of long‐term health condition (No (ref) yes any). Care profile: duration of care (< 1 year (ref) 1 to; years) > 3 to 5 years; 5+ years), and support of care recipient with palliative care (no (ref) yes). The models for ‘No services appropriate for cultural background', ‘No services appropriate for my gender identity', and ‘No services appropriate for my sexual identity' were considered unstable because the number of outcome events in at least one priority‐group category was fewer than five. Bold values are significant difference on *t*‐test/logistic regression/mediation analysis *p* < 0.05. For all other models, the number of outcome events exceeded 30 in each priority‐group category, and the estimated coefficients changed only slightly after excluding influential observations, supporting the stability of the model estimates. The Hosmer–Lemeshow goodness‐of‐fit tests for all models were not significant (*p* > 0.05), indicating adequate model calibration.

Carers from CALD backgrounds had significantly higher odds of reporting any barriers to accessing supportive care services for themselves compared to non‐priority group carers (aOR = 1.81 95% CI [1.03; 3.16] *p* = 0.038), as well as specific barriers including: long waiting periods (aOR = 1.77 95% CI [1.25; 2.52] *p* = 0.001), services not available when needed (aOR = 1.58 95% CI [1.10–2.26] *p* = 0.013), high cost of services making them unaffordable (aOR = 1.80 95% CI [1.21–2.69] *p* = 0.004), and to report services were not appropriate for their cultural background (aOR = 33.03 95% CI [12.18; 89.57] *p* < 0.001), gender identity (aOR = 11.14 95% CI [3.66; 33.93] *p* < 0.001), and sexual identity (aOR = 9.22 95% CI [2.64–32.25] *p* < 0.001).

No differences were found for accessing supportive care services for carers from any priority group compared to non‐priority group carers. Additionally, there were no significant differences in reporting barriers to accessing supportive care between LGBTIQA+ carers and carers who were not part of any priority group. The sensitivity analyses using multiple imputation (Supporting Information: Table S3), produced estimates similar to those from the complete‐case analyses (Table 3), supporting the robustness of the findings.

#### 3.4.1 Mediation Analysis

Mediation models examining whether health literacy mediated the relationship between priority group status (rural/remote vs non‐rural/remote, CALD vs non‐CALD, and LGBTIQA+ vs non‐LGBTIQA + ) and access to supportive care services, showed no significant indirect, direct, or total effects for any priority group for any type of supportive care use (Table 4).

**Table 4:** Mediation analysis of effect of carer priority population group via health literacy total score on (i) accessing support services (any, formal and informal), and (ii) reporting barriers to access.

	Rural/remote			CALD			LGBTIQA+		
	(*n *= 332)			(*n* = 324)			(*n* = 110)		
	ADjOR	[95% CI]	*p* value	ADjOR	[95% CI]	*p* value	ADjOR	[95% CI]	*p* value
*Supportive care use*									
Supportive care [any] accessed (yes)									
Natural indirect effect	1.01	[0.99; 1.04]	0.355	0.98	[0.90; 1.07]	0.687	0.99	[0.97; 1.03]	0.734
Naturel direct effect	1.06	[0.80; 1.41]	0.690	1.00	[0.69; 1.44]	0.998	0.66	[0.42; 1.01]	0.058
Total effect	1.08	[0.81; 1.43]	0.623	0.98	[0.69; 1.40]	0.918	0.65	[0.42; 1.01]	0.054
Formal supportive care (yes)									
Natural indirect effect	1.02	[0.98; 1.06]	0.282	1.01	[0.92; 1.11]	0.869	1.00	[0.96; 1.03]	0.815
Naturel direct effect	0.89	[0.67; 1.19]	0.436	1.45	[0.97; 2.17]	0.069	0.83	[0.52; 1.33]	0.442
Total effect	0.91	[0.68; 1.22]	0.517	1.46	[0.99; 2.16]	0.055	0.83	[0.52; 1.32]	0.432
Informal supportive care (yes)									
Natural indirect effect	0.99	[0.95; 1.02]	0.485	0.98	[0.85; 1.13]	0.784	1.01	[0.94; 1.08]	0.816
Naturel direct effect	1.24	[0.85; 1.79]	0.261	0.75	[0.44; 1.28]	0.290	0.70	[0.36; 1.38]	0.307
Total effect	1.22	[0.85; 1.77]	0.288	0.73	[0.44; 1.23]	0.240	0.71	[0.36; 1.40]	0.320
*Barriers to accessing supportive care services*									
Barriers [linear]									
Natural indirect effect	0.90	[0.75; 1.07]	0.216	**1.25**	**[1.06; 1.47]**	**0.007**	1.05	[0.79; 1.41]	0.720
Naturel direct effect	**1.55**	**[1.08; 2.24]**	**0.017**	1.55	[0.88; 2.73]	0.131	0.85	[0.49; 1.48]	0.565
Total effect	1.39	[0.94; 2.06]	0.099	**1.94**	**[1.09; 3.44]**	**0.024**	0.90	[0.48; 1.67]	0.731

*Note:* Models controling for: carer characteristics: age (18–49 (ref); 50–60 + ), gender (female (ref) male), employment (not in paid employment (ref), in paid employment), education (<=Year 12 (ref); certificate/diploma; Bachelor + ), presence of long‐term health condition (No (ref) yes any). Care profile: duration of care (< 1 year (ref) 1 to; years) > 3 to 5 years; 5+ years), and support of care recipient with palliative care (no (ref) yes). Bold values are significant difference on *t*‐test/logistic regression/mediation analysis *p* < 0.05.

Abbreviations: NIE, indirect effect; NDE, direct effect; total effect.

The model examining whether health literacy mediated the relationship between CALD status (CALD vs non‐CALD) and perceived barriers to accessing services, showed a significant total effect was observed (aOR = 1.94 95% CI [1.09; 3.44] *p* = 0.024). The indirect effect was also significant (aOR = 1.25 95% CI [1.06; 1.47] *p* = 0.007), while the direct effect was not significant, indicating full mediation. Results suggest health literacy rather than CALD status, may explain the increased likelihood of reporting barriers to accessing services among carers from CALD communities. In contrast, no significant mediation effects of health literacy were observed for rural/remote and LGBTIQA+ carers compared to their non‐priority counterparts and the relationship between reported barriers to accessing supportive care.

## 4 Discussion

To our knowledge, this is the first study to examine patterns of supportive care service use, perceived barriers to access, and the role of health literacy among more than 1,500 adult Australian cancer carers involving three priority groups.

Specifically, findings demonstrated inequities in access to supportive care among carers from priority populations, with particularly pronounced disparities for rural/remote and CALD carers. Rural and remote carers had substantially higher odds of experiencing barriers to accessing supportive care services, particularly structural and logistical barriers, such as local service unavailability and long waiting periods. These findings are consistent with previous research demonstrating the heightened burden experienced by rural and remote cancer carers [15, 32]. Although rural and remote carers reported greater access barriers, this relationship was not mediated by health literacy, showing opportunity to focus on addressing the structural barriers to improve cancer care, rather than individual factors. Supporting this interpretation, an Australian study examining support‐seeking behaviours of rural cancer carers found that support was typically sought from general practitioners or online sources [27]. However, carers in rural and remote areas frequently report difficulties accessing GP based care, including high workforce turnover in rural practices and longer waiting times [28, 33]. Reliance on online resources may be problematic given variability in the quality, accuracy, and applicability of available health information [34]. Collectively, these findings highlight the informational and practical challenges faced by carers in rural and remote Australia, aligning with existing evidence on structural barriers and the need for cross‐sector action to address equity gaps [35]. Given the persistent geographic inequities in cancer despite increasing availability of digital information [36], interventions focused exclusively on improving carers’ knowledge or confidence may be insufficient without concurrent investment in rural service infrastructure and workforce capacity.

Carers from CALD communities were identified as a group in need of further support, with poorer outcomes compared to other carer groups. They reported significantly more barriers in accessing supportive care services, showing a need to design and deliver appropriate and tailored programs to people from these communities. In addition to long waiting times, high cost, and service availability, CALD carers were markedly more likely to report that available services were not culturally inappropriate and insufficiently inclusive of their gender or sexual identity. While these findings should be interpreted with caution due to number of outcome events within models, findings align with recent Australian scoping review synthesising evidence from 64 studies [13], which identified multilevel barriers to health service access for CALD populations, particularly, structural and policy‑level barriers. The review highlighted systemic inadequacies in service design and delivery, including limited cultural responsiveness and insufficient capacity of health systems to address the complex needs of CALD communities. Consistent with this broader literature, Australian studies of people with cancer and carers from CALD communities highlight opportunities to enhance service responsiveness, with language accessibility, health system navigation, and support for functional and interactive health literacy identified as key areas for improving engagement with cancer care and supportive services [37, 38].

Carers identifying as LGBTIQA+ did not differ significantly from non‐priority groups in their access of supportive care services or in perceived barriers to accessing supportive care. While these findings may suggest more equitable access, when considered alongside well documented experiences of stigma and discrimination within healthcare settings [39, 40], barriers to care may still exist. In addition, the findings from our study may reflect that supportive care services may have become more inclusive over time, particularly within cancer care settings that have prioritised equity and diversity initiatives [41]. Secondly, the relatively small sample size of LGBTIQA+ carers may have limited statistical power to detect differences. Lastly, barriers experienced by LGBTIQA+ carers may be more closely related to quality of care, psychological safety, disclosure of identity, or experiences of discrimination and stigma than to service access itself [42, 43, 44]. These dimensions were not captured in the present study and warrant further investigation.

Notably, health literacy fully accounted for the association between CALD status and perceiving barriers to accessing supportive care, indicating that lower health literacy represented a key mechanism through which these disparities are produced. Health literacy is increasingly understood as a relational concept that depends on the responsiveness of organisations as well as the skills of individuals [45]. For CALD carers, language barriers, unfamiliarity with Australian health services, and limited availability of culturally adapted information may reduce opportunities to identify, understand, and engage with supportive care, even where these services formally exist [46]. Together, these associations highlight how supportive care services often fail to meet the linguistic, cultural, and identity related needs of CALD carers. Addressing this gap requires the development of culturally responsive, co‐designed support models that improve service acceptability, reduce stress, and enable meaningful engagement.

Taken together, the findings suggest that inequities in supportive care access arise through different mechanisms across priority populations. For rural and remote carers, barriers appear predominantly structural, reflecting limitations in service availability and accessibility. For CALD carers, disparities appear to arise through both structural factors and challenges related to navigating and engaging with services, as reflected in the mediating role of health literacy. These findings indicate that a single equity strategy is unlikely to be effective across diverse populations; rather, tailored approaches are required that address the specific mechanisms through which inequities emerge.

### 4.1 Implications (Clinical and Research)

Findings highlight the need for tailored, equity‐focused supportive care interventions to support cancer carers, particularly those from priority populations. Strategies should address both health literacy and systemic access barriers, prioritising improvement in service availability and coordination, particularly in rural and remote areas, and generate and embed co‐designed culturally safe, inclusive, and affordable support models. Critically, implementation research is needed to identify how best to deliver, integrate, and sustain these interventions in real‐world settings, while rigorous evaluation is required to assess their reach, efficacy, cost‐effectiveness and equity impacts. Future research could further explore the intersection of gender and priority population characteristics, as gender may influence both supportive care needs and access to services among cancer carers overall and among priority groups [47, 48]. Understanding these intersecting factors may help inform more tailored and equitable supportive care interventions.

At a policy level, the findings support ongoing implementation of Australia's National Cancer Plan [46] and broader health equity priorities by highlighting the need for supportive care systems that are responsive to the needs of diverse carers. Importantly, similar to patients, policies should recognise carers as distinct populations within cancer care and ensure that supportive care programs are designed, funded, and evaluated with carers' needs explicitly considered.

### 4.2 Study Limitations

Participants were recruited through a non‐probabilistic sample across two distinct approaches, which may have influenced sample characteristics. However, given the absence of any national registry of informal cancer carers, this approach allowed us to generate a substantial and meaningful dataset that captured the diversity and experiences of these often‐under‐represented groups. Cross‐sectional design and reliance on self‐report measures may introduce bias and limit causal interpretations. A limitation of this study was the proportion of participants excluded from regression analyses due to missing outcome data. Participants excluded from analyses differed from included participants on some characteristics; including CALD status, age, education, care duration and provision of palliative care support. However, findings from multiple imputation sensitivity analyses were consistent with those from the complete‐case analyses, indicating that missing data were unlikely to have substantially influenced the study findings. In addition, as analyses were exploratory in nature we did not adjust for multiple testing. Therefore, findings should be interpreted with appropriate caution due to possibility of Type I error. Due to power constraints, the CALD group was not disaggregated into specific cultural subgroups, and data on the number of years participants had lived in Australia were not collected, preventing differentiation among CALD carers.

## 5 Conclusion

This study highlights limited access to supportive care services among cancer carers, in combination with a high prevalence of perceived systemic barriers to accessing care. Inequities in supportive care access persist, particularly for rural/remote and CALD population groups. Collectively, the findings support the need for co‐designed, accessible, inclusive supportive care interventions and systems that address health literacy and systemic access barriers, that are responsive to geographic, cultural, linguistic and social diversity. Addressing these gaps will improve equity in supportive care access and outcomes for informal cancer carers and the people they support.

## Author Contributions

**Stephanie P. Cowdery:** conceptualisation, investigation, writing – original draft, methodology, writing – review and editing, formal analysis, data curation, project administration, validation. **Patricia M. Livingston:** conceptualisation, funding acquisition, writing – review and editing, supervision, methodology. **Thach Tran:** writing – review and editing, formal analysis, validation. **Antonina Mikocka‐Walus:** writing – review and editing. **Anna Ugalde:** writing – review and editing. **Eva Yuen:** writing – review and editing. **Hannah Jongebloed:** writing – review and editing. **Natalie Winter:** writing – review and editing. **Nicole Kiss:** writing – review and editing. **Nikki McCaffrey:** writing – review and editing. **Norah Refahi:** writing – review and editing. **Victoria M. White:** conceptualisation, funding acquisition, methodology, writing – review and editing; supervision.

## Conflicts of Interest

The authors declare no conflicts of interest.

## Supporting information


**Table S1:** Characteristics of carers included versus excluded (*n* = 1,536).
**Table S2:** Numbers and proportion of missing data by variables included in the models in the analysis sample (*n* = 1,154).
**Table S3:** Logistic regression: full list of controlling factors + missing data treated using multiple imputation.

## Data Availability

Psycho‐Oncology (PON)The data that support the findings of this study are not publicly available due to privacy and ethical restrictions. De‐identified data may be made available from the corresponding author upon reasonable request, subject to institutional ethics approval.

## Footnotes

1 Website: https://www.loteagency.com.au/.
