# Supplementary material for: Access to Supportive Care Among Australian Cancer Carers: Experiences of Priority Populations
Source: Health Expect. 2026 Sep 9;29(5):e70830. doi: 10.1111/hex.70830 (PMC13559169; doi:10.1111/hex.70830)
Supplement: Supplementary file 1 [file HEX-29-e70830-s001.docx]

Supplementary Table 1. Characteristics of carers included versus excluded (n▒=▒1,536)

|  | **Included**  **(n**▒=▒**1,154)** | | **Excluded**  **(n**▒=▒**382)** | |
| --- | --- | --- | --- | --- |
|  | n | % | n | % |
| **Carer characteristics** |  |  |  |  |
| Priority group |  |  |  |  |
| Rural/Remote (yes) | 332 | 28.8 | 113 | 29.6 |
| CALD (yes)*** | 324 | 28.1 | 68 | 17.8 |
| LGBTIQA+ (yes) | 110 | 9.5 | 25 | 6.5 |
| Age** |  |  |  |  |
| 18-49 years | 429 | 37.2 | 106 | 27.7 |
| 50-59 years | 321 | 27.8 | 111 | 29.1 |
| 60+ years | 404 | 35.0 | 165 | 43.2 |
| Gender |  |  |  |  |
| Female | 947 | 82.1 | 276 | 72.3 |
| Male | 193 | 16.7 | 39 | 10.2 |
| Employment |  |  |  |  |
| Not in paid employment | 488 | 42.3 | 144 | 37.7 |
| Employed (current) | 662 | 57.4 | 174 | 45.5 |
| Education*** |  |  |  |  |
| Year 12 certificate or less | 193 | 16.7 | 81 | 21.2 |
| Certificate/diploma | 328 | 28.4 | 97 | 25.4 |
| Bachelor’s degree or higher | 604 | 52.3 | 130 | 34.0 |
| Presence of long-term health condition (yes) | 484 | 41.9 | 149 | 39.0 |
| **Care profile** |  |  |  |  |
| Duration of care** |  |  |  |  |
| Less than 1 year | 406 | 35.2 | 155 | 40.6 |
| More than 1 year but less than 3 | 473 | 41.0 | 134 | 35.1 |
| More than 3 years but less than 5 | 208 | 18.0 | 53 | 13.9 |
| 5 or more years | 67 | 5.8 | 40 | 10.5 |
| Support of care recipient with palliative care (yes)* | 441 | 38.2 | 121 | 31.7 |

*p▒<▒0.05 on chi-square

**p▒<▒0.01 on chi square

***p▒<▒0.001 on chi square

Supplementary Table 2. Numbers and proportion of missing data by variables included in the models in the analysis sample (n▒=▒1,154)

|  | **Missing data** | |
| --- | --- | --- |
|  | n | % |
| **Carer characteristics** |  |  |
| Priority group |  |  |
| Rural/Remote | 20 | 1.7 |
| CALD | 1 | 0.1 |
| LGBTIQA | 2 | 0.2 |
| Age | 0 | 0.0 |
| Gender | 14 | 1.2 |
| Employment | 4 | 0.3 |
| Education | 29 | 2.5 |
| Presence of long-term health condition (yes) | 31 | 2.7 |
| Duration of care | 0 | 0.0 |
| Support of care recipient with palliative care (yes)* | 0 | 0.0 |
| **Supportive care use** |  |  |
| Supportive care [any] accessed (yes) | 0 | 0.0 |
| Formal supportive care (yes) | 0 | 0.0 |
| Informal supportive care (yes) | 0 | 0.0 |
| **Barriers to accessing supportive care services** |  |  |
| Barriers [any] | 4 | 0.3 |
| Long waiting periods before you can access services | 4 | 0.3 |
| Services not being available locally | 0 | 0.0 |
| Services not available when I needed them | 0 | 0.0 |
| No services appropriate for cultural background | 0 | 0.0 |
| No services appropriate for my gender identity | 0 | 0.0 |
| No services appropriate for my sexual identity | 0 | 0.0 |
| High cost of services mean they are unaffordable | 0 | 0.0 |
| Finding information about services available to me is difficult | 0 | 0.0 |
| Time and energy needed to organise services | 0 | 0.0 |
| Lack of time to seek out support or services for myself | 0 | 0.0 |

Supplementary Table 3. Logistic regression: full list of controlling factors + missing data treated using multiple imputation

|  | Rural/remote | | | CALD | | | LGBTIQA+ | | |
| --- | --- | --- | --- | --- | --- | --- | --- | --- | --- |
|  | (n▒=▒445) | | | (n▒=▒392) | | | (n▒=▒135) | | |
|  | ADjOR | [95% CI] | pvalue | ADjOR | [95% CI] | pvalue | ADjOR | [95% CI] | pvalue |
| **Supportive care use** |  |  |  |  |  |  |  |  |  |
| Supportive care [any] accessed (yes) | 1.09 | [0.83; 1.44] | 0.524 | 0.89 | [0.61; 1.28] | 0.513 | 0.72 | [0.47; 1.11] | 0.138 |
| Formal supportive care (yes) | 0.87 | [0.65; 1.17] | 0.361 | 1.47 | [1.00; 2.17] | 0.052 | 0.94 | [0.57; 1.54] | 0.806 |
| Informal supportive care (yes) | 1.21 | [0.84; 1.73] | 0.300 | 0.78 | [0.49; 1.25] | 0.294 | 0.73 | [0.38; 1.42] | 0.354 |
| **Barriers to accessing supportive care services** |  |  |  |  |  |  |  |  |  |
| Barriers [any] | 1.34 | [0.89; 2.80] | 0.165 | 1.62 | [0.96; 2.73] | 0.069 | 0.97 | [0.50; 1.89] | 0.937 |
| Long waiting periods before you can access services | 1.39 | [1.07; 1.80] | 0.014 | **1.82** | **[1.35; 2.45]** | **<0.001** | 1.11 | [0.75; 1.65] | 0.599 |
| Services not being available locally | **3.82** | **[2.95; 4.95]** | **<0.001** | 1.32 | [0.96; 1.82] | 0.093 | 0.88 | [0.57; 1.36] | 0.562 |
| Services not available when I needed them | **1.52** | **[1.17; 1.98]** | **0.002** | **1.55** | **[1.14; 2.11]** | **0.006** | 0.89 | [0.59; 1.35] | 0.586 |
| No services appropriate for cultural background | 0.84 | [0.35; 2.04] | 0.703 | **36.22** | **[13.75; 95.42]** | **<0.001** | 1.12 | [0.49; 2.54] | 0.787 |
| No services appropriate for my gender identity | 0.93 | [0.33; 2.63] | 0.894 | **9.20** | **[3.61; 23.43]** | **<0.001** | 1.25 | [0.46; 3.45] | 0.662 |
| No services appropriate for my sexual identity | 0.71 | [0.19; 2.63] | 0.612 | **6.66** | **[2.46; 18.03]** | **<0.001** | **3.77** | **[1.52; 9.30]** | **0.004** |
| High cost of services mean they are unaffordable | 1.07 | [0.77; 1.49] | 0.686 | **1.85** | **[1.31; 2.62]** | **0.001** | 1.16 | [0.74; 1.83] | 0.513 |
| Finding information about services available to me is difficult | 0.97 | [0.74; 1.27] | 0.800 | 0.88 | [0.63; 1.22] | 0.441 | 1.01 | [0.66; 1.56] | 0.955 |
| Time and energy needed to organise services | 1.28 | [1.00; 1.63] | 0.053 | 0.83 | [0.62; 1.12] | 0.222 | 1.11 | [0.75; 1.62] | 0.611 |
| Lack of time to seek out support or services for myself | 1.24 | [0.97; 1.59] | 0.090 | 0.89 | [0.66; 1.21] | 0.453 | 1.28 | [0.87; 1.87] | 0.211 |

Models controling for: Carer characteristics: age (18-49 (ref); 50-59; 60▒+▒), gender (female (ref) male), employment (not in paid employment (ref), in paid employment), education (<=Year 12 (ref); certificate/diploma; Bachelor▒+▒), presence of long-term health condition (No (ref) yes any). Care profile: duration of care (▒<▒1 year (ref); 1-▒<▒3 years) 3-▒<▒5 years; 5+ years), and support of care recipient with palliative care (no (ref) yes).
